# Photocatalytic Membrane Reactor for the Removal of C.I. Disperse Red 73

**DOI:** 10.3390/ma8063633

**Published:** 2015-06-18

**Authors:** Valentina Buscio, Stephan Brosillon, Julie Mendret, Martí Crespi, Carmen Gutiérrez-Bouzán

**Affiliations:** 1 Institut d’Investigació Tèxtil i Cooperació Industrial de Terrassa (INTEXTER), Universitat Politècnica de Catalunya-BarcelonaTech (UPC). C/Colom 15, Terrassa 08222, Spain; E-Mails: valentina.buscio@intexter.upc.edu (V.B.); crespi@etp.upc.edu (M.C.); 2 Institut Européen des Membranes (IEM), UMR Université Montpellier 2 Place E. Bataillon, Montpellier F-34095, France; E-Mails: Stephan.Brosillon@univ-montp2.fr (S.B.); Julie.Mendret@univ-montp2.fr (J.M.)

**Keywords:** photocatalytic membrane reactor, titanium dioxide, C.I. Disperse Red 73, UV light, polysulfone membrane, microfiltration

## Abstract

After the dyeing process, part of the dyes used to color textile materials are not fixed into the substrate and are discharged into wastewater as residual dyes. In this study, a heterogeneous photocatalytic process combined with microfiltration has been investigated for the removal of C.I. Disperse Red 73 from synthetic textile effluents. The titanium dioxide (TiO_2_) Aeroxide P25 was selected as photocatalyst. The photocatalytic treatment achieved between 60% and 90% of dye degradation and up to 98% chemical oxygen demand (COD) removal. The influence of different parameters on photocatalytic degradation was studied: pH, initial photocatalyst loading, and dye concentration. The best conditions for dye degradation were pH 4, an initial dye concentration of 50 mg·L^−1^, and a TiO_2_ loading of 2 g·L^−1^. The photocatalytic membrane treatment provided a high quality permeate, which can be reused.

## 1. Introduction

Synthetic dyes are widely used in the textile industry. Approximately 800,000 tonnes of dyes are produced annually worldwide [1]. However, about 15% of the dyes consumed are lost in the textile effluents [2]. Textile wastewater is characterized by its high chemical oxygen demand (COD), salinity, variable pH values, and high temperature [3,4,5,6]. In addition, the presence of a very low concentration of dyes in wastewater is highly visible [7].

The dyes are classified according to how they are applied in the dyeing process. The most common dyes are reactive, acid, and disperse. Reactive dyes are water-soluble, mainly applied to cotton. Acid dyes are water-soluble and applied from an acidic solution to nylon, wool, silk, and some modified acrylic textiles. Finally, disperse dyes are used for polyesters fibers. They have very low water solubility, so they are applied as a dispersion in the dyeing process [8].

Many processes have been studied to treat textile wastewater in order to remove color. The most used treatments are physical and chemical techniques such as coagulation-flocculation, adsorption, or membrane processes. Nonetheless, these treatments do not destroy the dye and a post-treatment, such as incineration, is required [9].

In recent years, the interest for advanced oxidation processes (AOPs) has increased, especially heterogeneous photocatalytic processes. Titanium dioxide (TiO_2_) is the most used photocatalyst due to its good photocatalytic activity, nontoxicity, chemical inertness, and low cost [10].

The heterogeneous photocatalytic process starts when a semiconductor absorbs photons whose energy is equal to or greater than its band gap (e.g., = 3.2 eV for TiO_2_). This adsorption enables the promotion of an electron (e^−^) from the valence band (vb) of the semiconductor to the conduction band (cb), generating holes (h^+^) in the valence band [11]. The recombination of the electron and the hole must be prevented.

The electron can reduce the organic species or react with the O_2_, which is either adsorbed on the semiconductor surface or dissolved in water, reducing it to a superoxide radical anion (O_2_^−**•**^). The hole can oxidize the organic molecule or react with OH^−^ or H_2_O, oxidizing them into OH**^•^** radicals [12].

The highly oxidant species generated during the photocatalytic process are responsible for the photodegradation of organic substrates such as dyes. The process can be expressed according to Equations (1)–(8) [11,13]:

TiO_2_ + hv(UV) → TiO_2_ (e_CB_^−^ + h_VB_^+^) (1)

TiO_2_(h_VB_^+^) + H_2_O → TiO_2_ + H^+^ + OH**^•^** (2)

TiO_2_(h_VB_^+^) + OH^−^ → TiO_2_ + OH**^•^** (3)

TiO_2_(e_VB_^−^) + O_2_ → TiO_2_ + O_2_^−**•**^ (4)

O_2_^−**•**^ + H^+^ → HO_2_**^•^** (5)

Dye + OH**^•^** → degradation products (6)

Dye + h_VB_^+^ → oxidation products (7)

Dye + e_VB_^−^ → redeuction products (8)

The oxidation species responsible for organic substrate degradation depends on experimental conditions (pH, pollutant, concentration, *etc.*). The dominant oxidative species can be identified adding different species quenchers [14].

The main limitation of the photocatalytic process is the recovery of the photocatalyst from the solution. This problem can be solved by doing a coupling of photocatalysis with membrane processes [15]. In addition, this coupling enables the confinement of the photocatalyst in the reaction environment, the control of the residence time of the molecule in the reactor by means of the transmembrane flux, and the achievement of a continuous process with simultaneous photocatalyst and product separation from the reaction environment. Heterogeneous photocatalytic oxidation may be combined with different membrane processes, such as microfiltration (MF) [16,17,18,19], ultrafiltration (UF) [20,21,22], nanofiltration (NF) [23,24], and direct contact membrane distillation (DCMD, MD) [10,25,26]. Photocatalytic membrane reactors (PMRs) described in the literature can be divided into two groups: (I) reactors with photocatalysts suspended in a feed solution and (II) reactors with photocatalysts supported on the membrane [27]. A comparison between PMRs with a photocatalyst in suspension and with a photocatalyst supported on a membrane on the purification of water contaminated with dyes was reported by Grzechulska *et al.* [28]. The authors studied the degradation of three dyes: C.I. Acid Red 18, C.I. Acid Yellow 36, and C.I. Direct Green 99. Results showed that the time of discoloration was shorter in the suspended system than in case of the supported photocatalyst.

Several studies with photocatalysts in suspension have been reported to treat different solutions containing dyes. In general, color removal of reactive dyes [10,29] and acid dyes [16,30] has been widely investigated, but fewer studies have been carried out on disperse dyes despite the fact that they are used for dyeing polyester fibers, which are the most consumed fibers in the world. Due to the high worldwide consumption of polyester, the interest for the disperse dyes and their associated environmental problems has increased in recent years [31]. It is important to highlight that after the dyeing process, the dye is not totally adsorbed by polyester and it is discharged into wastewater. A proper removal of disperse dye in the effluent would allow the water to be reused, which is an important advantage from the economic and environmental points of view.

Taking into account these considerations, this paper investigates the potential use of a photocatalytic membrane reactor for the degradation of C.I. Disperse Red 73 (DR73) using TiO_2_ in suspension as a photocatyst. In order to optimize the process, the effect of several parameters such as pH, initial dye concentration, and TiO_2_ loading on photodegradation efficiency was tested. As far as we know, no studies based on the degradation of disperse dyes by means of photocatalytic membrane reactor have been carried out.

## 2. Experimental Section

### 2.1. Reagents

The photocatalyst used in this study was commercial TiO_2_ powder (Sigma-Aldrich). The average diameter of TiO_2_ particles was 21 nm and BET was 50 ± 15 m^2^·g^−1^.

**Figure 1:**
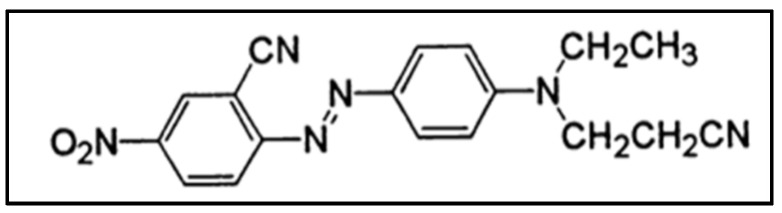
Chemical structure of Disperse Red 73 (DR73).

C.I. Disperse Red 73 was supplied by Archroma (Figure 1). Its molecular weight is 348 g·moL^−1^.

To determine the dye concentration, *N*,*N*-dimethylformamide (purity 99%, Merck) and deionised water were employed as solvents.

The pH of synthetic effluents containing DR73 was adjusted with 1 M solutions of NaOH or HCl.

### 2.2. Photocatalytic Membrane Experiments

**Figure 2:**
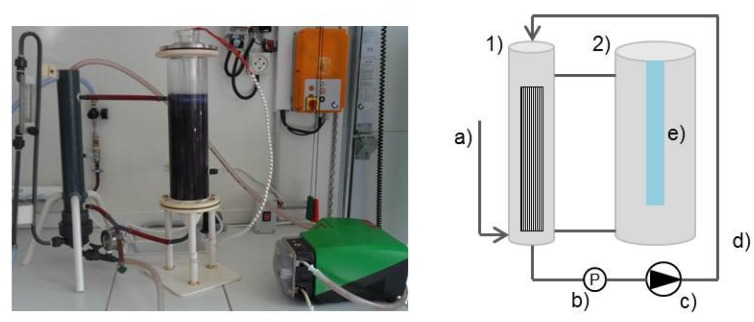
Photocatalytic membrane reactor: (**1**) membrane reactor; (**2**) photocatalytic reactor; (**a**) air inlet; (**b**) pressure measurement; (**c**) peristaltic pump; (**d**) permeate; and (**e**) UV lamp.

The photocatalytic membrane reactor was equipped with a photocatalytic reactor (2.2 L) and an outside membrane module (0.3 L) (Figure 2).

A hollow fiber membrane module manufactured by Polymem Company (Toulouse, France) was used. Its main specifications and operation characteristics are shown in Table 1.

**Table 1:** Membrane characteristics.

Material	Polysulfone
Nominal pore size (µm)	0.2
External diameter (mm)	1.4
Fiber length (cm)	32
Surface area (m^2^)	0.3
Hydraulic resistance (m^−1^)	4·1011
Pure water permeability at 20 °C (L·h^−1^·m^−2^·bar)	227

Air was injected at a flow rate of 200 L·h^−1^ through a diffuser placed at the bottom of the membrane module. This injection of air enables an air lift and then the liquid goes from the membrane module to the photoreactor. This circulation allowed the homogenization of the solution.

A peristaltic pump (Watson-Marlow) was used to fix the permeate flux at 20 L·h^−1^·m^−2^ and to recirculate the permeate to the membrane module.

The light source was a 24 W UV lamp PL-L 24 W/10/4P (Philips, Amsterdam, The Netherlands), with a wavelength of 365 nm, located in a quartz vessel in the centre of the photocatalytic reactor.

To simulate the effluents discharged after the dyeing process, solutions of DR73 were prepared in deionised water. Before photodegradation, the dye-TiO_2_ mixture was kept for 30 min in the dark in order to allow adsorption of the dye molecules on the TiO_2_ surface. The effects of different parameters on the process were tested. Photocatalytic experiments were conducted at different pH levels (4, 6, and 10), different amounts of TiO_2_ (between 0.5 and 2 g·L^−1^), and different initial dye concentrations (50, 75, and 100 mg·L^−1^).

### 2.3. Analytical Methods and Measurements

The membrane fouling was evaluated by recording the ∆P and measuring the normalized water permeability with Equation (9).

Lp(20 °C) = J/∆P · µ(20 °C)/µT
(9)

where J is the permeate flux (m^3^·m^−2^·s^−1^), ΔP is the transmembrane pressure (Pa), µ (20 °C) is the viscosity of the fluid at 20 °C (Pa·s), and µ_T_ is the viscosity of the fluid at working temperature (Pa·s).

Samples were taken from the photocatalytic reactor and then centrifuged with a Sigma 3–16 k centrifuge in order to separate the TiO_2_ particles from the solution.

Dye removal (%R_dye_) was calculated from concentrations of dye in the feed and the photocatalytic reactor using Equation (10):

%R_dye_ = ((C_f_ − C_t_)/C_f_)·100 (10)

where C_f_ and C_t_ are the concentrations of dye in the feed and the photocatalytic reactor at time t, respectively. The dye was dissolved in a solution of water/*N*,*N*-dimethylformamide (1/1, *v/v*), and its concentration was determined with a UV-Vis spectrophotometer UV-2401 (Shimadzu Corporation, Kyoto, Japan) at the maximum wavelength of the visible spectrum (λ_max_ = 528 nm).

COD was determined according to the method 5220 D recommended by American Public Health Association [32]. The COD reduction (%R_COD_) was calculated using Equation (11):

%R_COD_ = ((COD_f_ − COD_p_)/COD_f_)·100 (11)

where COD_f_ and COD_p_ are the COD values in the feed and permeate, respectively.

The pH was determined according to the method 4500 H^+^ B [32] using a pH meter Ion 510 (Eutech Instruments, Landsmeer, The Netherlands).

## 3. Results and Discussion

### 3.1. Previous Studies

**Figure 3:**
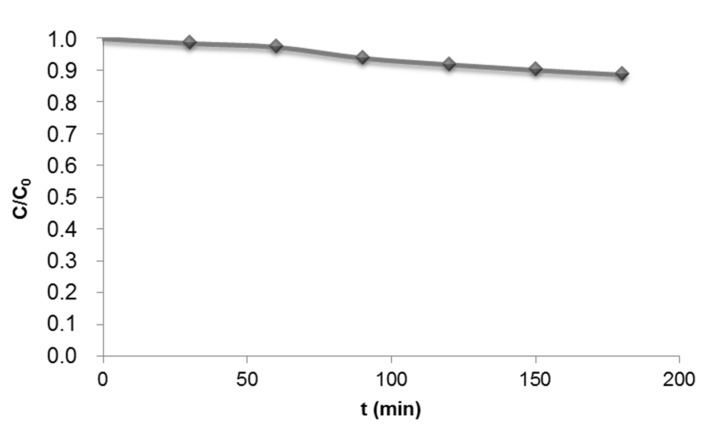
DR73 degradation after 180 min of UV irradiation (λ = 365 nm).

Some dyes can be degraded under UV irradiation without a photocatalyst. Before studying the photocatalytic degradation of DR73, the stability of the dye under UV light was tested (Figure 3).

No significant dye degradation was observed after 180 min of UV irradiation. It is important to highlight that certain disperse dyes are generally characterized by their high light fastness [33]. In the case of DR73 dye, its light fastness is 6 (value 1 indicates very low fastness and 8 corresponds to high fastness).

### 3.2. Photocatalytic Degradation of DR73

#### 3.2.1. Effect of Initial pH

The pH affects both the surface properties of the photocatalyst and the physical properties of dye solution, such as dye aggregation or dispersion. The effect of the pH was studied using a solution of 75 mg·L^−1^ of DR73 and a TiO_2_ dosage of 1 g·L^−1^.

**Figure 4:**
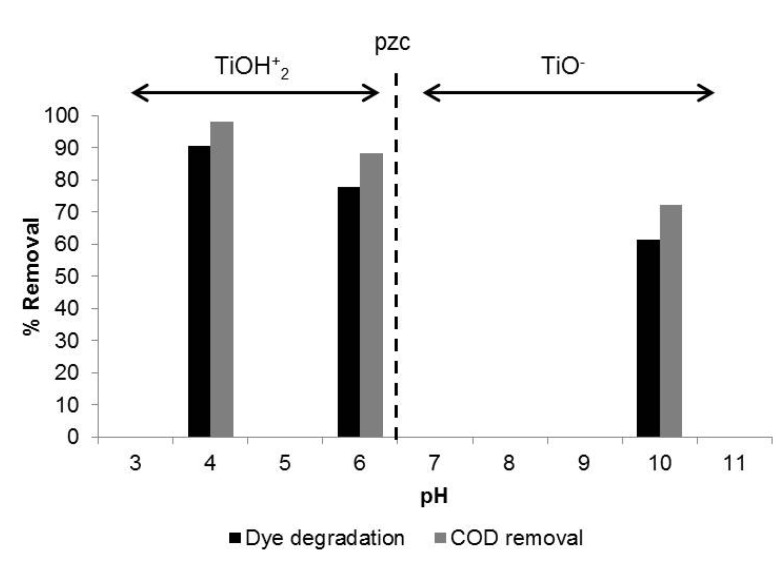
Influence of pH solution on the degradation of DR73.

Comparing experiments carried out at different pH values (4, 6, and 10), it can be observed that, after 180 min of UV irradiation, the highest dye degradation (90%) was achieved at pH 4, whereas that at pH 10, only 61% of dye degradation was obtained (Figure 4). The point of zero charge (pzc) of the TiO_2_ Aeroxide P25 is reported to be at pH 6.5. Thus, the TiO_2_ surface is positively charged in an acidic medium (pH < 6.5) and it is negatively charged in an alkaline medium (pH > 6.5), as is shown in Equations (12) and (13) [34].

When pH < pzc: TiOH + H^+^ ↔ TiOH_2_^+^ (12)

When pH > pzc: TiOH + OH^−^ ↔ TiO^−^ + H_2_O (13)

At pH lower than 6.5, the TiO_2_ is positively charged and attracts the negatively charged species from the solution, facilitating their photodegradation. The dye studied in this project is negatively charged in water solution.

Although the mechanism of degradation was not the aim of this work, several studies [11,14] have reported that, in acidic medium, holes are the major oxidation species, whereas at high pH, hydroxyl radicals are considered the predominant species.

As expected, the COD removal followed the same trend as the degradation of dye. About 98% of COD removal was obtained at pH 4, 88% at pH 7, and 72% at pH 10.

Several authors [35,36] reported that in very acidic medium, the strong adsorption of the dye particles on the TiO_2_ surface could reduce the active centres on the photocatalyst surface. On the other hand, in alkaline medium, the dye particles are hardly adsorbed on the catalyst surface. In addition, the dyeing process with disperse dyes is carried out at a pH between 5.5 and 6.5 [33]. A change of pH implies an increase in the conductivity of the effluents, which is one of the main problems of textile wastewater. For these reasons, pH 6 was selected for the subsequent studies.

#### 3.2.2. Effect of Photocatalyst Loading

To determine the influence of the photocatalyst loading in the process, a solution of 75 mg·L^−1^ at pH 6 was used.

**Figure 5:**
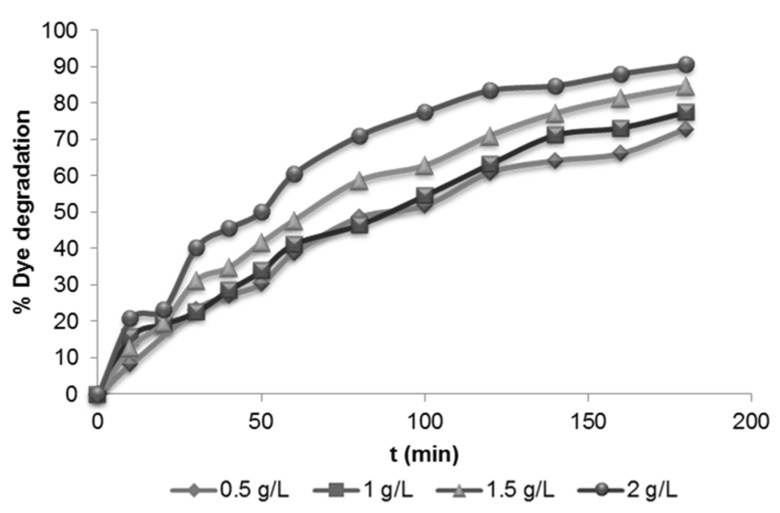
Evolution of the dye degradation for different TiO_2_ loading (initial dye concentration = 75 mg·L^−1^).

As can be observed in Figure 5, the COD removal ranged from 70% to 90% for the tested concentration of TiO_2_. The photocatalyst loading of 0.5 g·L^−1^ and 1 g·L^−1^ provided similar dye degradation (about 75%). However, when 2 g·L^−1^ of TiO_2_ was used, a remarkable increase of the dye degradation (90%) was observed.

Nonetheless, it is important to highlight that an excess of photocatalyst can produce a light screening, which causes a reduction in the surface area exposed to irradiation and thus could reduce the photocatalytic efficiency of the process [37].

Taking into account these considerations and to prevent membrane fouling, 1 g·L^−1^ of TiO_2_ was selected for subsequent experiments.

#### 3.2.3. Effect of Initial Dye Concentration

**Figure 6:**
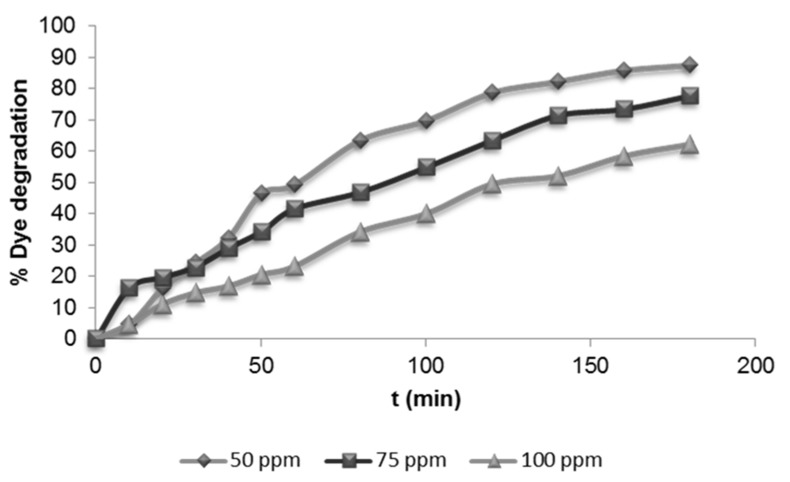
Influence of the initial dye concentration on dye degradation (1 g·L^−1^ TiO_2_).

The effect of the initial dye concentration was tested at a constant TiO_2_ dosage (1 g·L^−1^) and pH 6, with dye concentrations ranging from 50 to 100 mg·L^−1^. As can be observed in Figure 6, the dye degradation decreased (from 87% to 62%) when the dye concentration increased.

The higher the dye concentration, the higher the adsorption of the dye on the photocatalyst surface, which produced a decrease of OH^−^ adsorption and, consequently, diminished the formation of OH**^•^** radical, the principal oxidant in the photocatalysis process [38]. In addition, at a high dye concentration, the UV light might be absorbed by dye instead of the TiO_2_ particles. Similar results have been reported for the photocatalytic degradation of reactive [29,39] and direct dyes [40,41] and pharmaceutical compounds [42,43]. Liang *et al.* [42] observed that the photodegradation reached a saturation limit at high reactant concentration.

**Figure 7:**
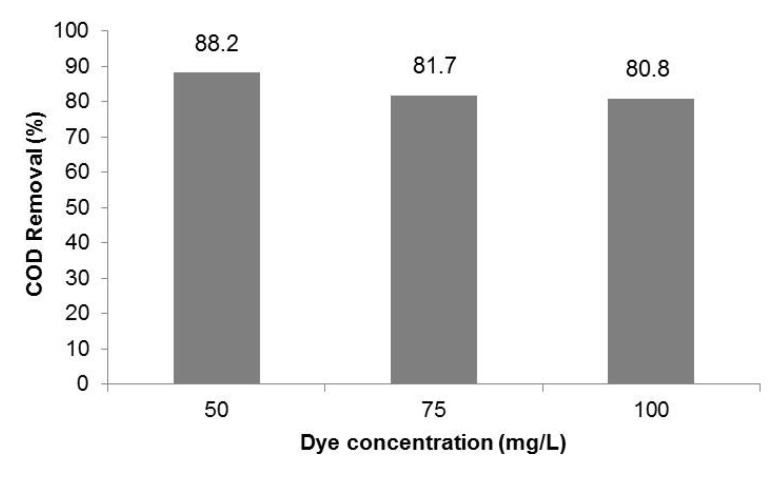
Influence of the dye concentration on COD removal (1 g·L^−1^ TiO_2_).

Regarding the COD removal (Figure 7), all experiments showed COD decrease higher than 80%.

The effect of the initial concentration of dye on the photocatalytic process can be described by a pseudo-first-order kinetic model with respect to the dye concentration. In general, photocatalytic degradation has a kinetic model according to Langmuir-Hinshelwood [15,16]:

R = dC/dt = −kC
(14)

**Figure 8:**
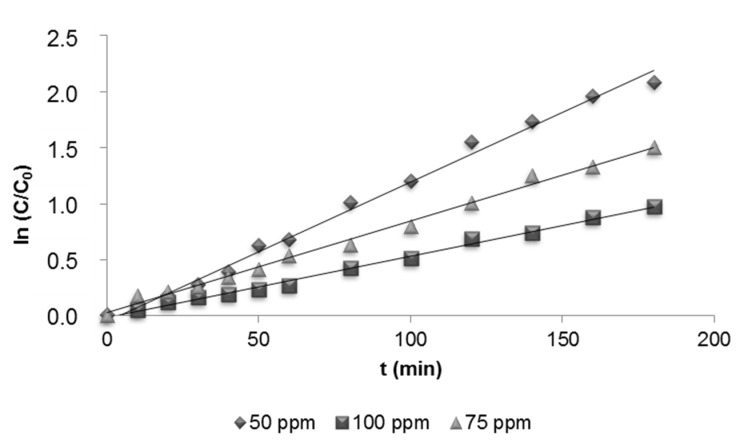
Relationship between ln (C/C_0_) and treatment time (t = 180 min and 1 g·L^−1^ TiO_2_).

Integrating Equation (14) with respect to time t, it can be simplified to the pseudo-first-order kinetic model Equation (15):

ln (C_0_/C_t_) = k_app_·t (15)

where, dC/dt is the rate of dye degradation (mg·L^−1^·min^−1^), C_0_ and C_t_ are initial concentration and concentration at time t of the dye (mg·L^−1^), respectively, and k_app_ is the degradation kinetic rate (min^−1^) [44]. The k_app_ was calculated from the slope of logarithmic concentration values *versus* time of treatment (Figure 8).

The results indicated that the degradation of DR73 fitted first-order kinetics and it followed the Langmuir-Hinshelwood model (Table 2). The value of k_app_ decreased with the increasing of the dye concentration.

**Table 2:** The pseudo-first-order degradation rate constants of DR73 at different initial dye concentrations and 1 g·L^−1^ TiO_2_.

Dye Concentration (mg·L^−1^)	K_app_ (min^−1^)	r^2^
50	0.0124	0,9937
75	0.0082	0,9934
100	0.0055	0,9943

### 3.3. Photocatalytic Membrane Treatment

#### 3.3.1. Permeate Quality

A synthetic effluent containing 75 mg·L^−1^ DR73 and 119 mgO_2_·L^−1^ COD was treated by means of a photocatalytic membrane reactor. The characterization of the permeate after 180 min of treatment showed dye concentrations lower than 0.5 mg·L^−1^ and COD values of about 10 mg·L^−1^. A full mineralization of the dye would probably be achieved with longer treatment, but with high associated cost. However, our previous studies [45] have shown that permeates with higher residual organic matter content are able to be successfully reused in new dyeing processes with disperse dyes. Due to the high water consumption in the textile industry, water reuse is an important challenge with advantages from both economical and environmental points of view.

#### 3.3.2. Effect of Photocatalytic Treatment on Membrane Fouling

The maintenance of the membrane is also an important point to take into account for the industrial application of the technology.

Generally, a significant fouling is observed when the photocatalysis process is combined with pressure-driven membrane processes, such as MF and UF [46,47], which results in either a flux decrease or a ∆P increase. To establish the factor affecting the membrane fouling, the ∆P was recorded with three effluents: pure water, water containing 1 g·L^−1^ of TiO_2,_ and a dye solution of 75 mg·L^−1^.

It was observed that when the solution with a photocatalyst was treated, the pressure increased with respect to the ∆P obtained with pure water, although it remained constant during the whole experiment. Thus, the presence of the photocatalyst produced an initial fouling on the membrane that did not increase along the experiment. According to Damodar *et al.* [29], this result could indicate that the TiO_2_ particles formed a very porous cake layer on the membrane surface. Finally, when the dye solution was treated, an increase of pressure was observed during the experiment indicating the formation of a dye deposit.

During the photocatalytic treatment, the pH was found to be the most influent parameter on the membrane fouling. At pH 10, an important increase of the ∆P was observed, whereas at pH 4, the fouling decreased. The high pH could influence the stability of the dye dispersion, producing an agglomeration of the dye particles whose size has an influence on fouling.

The efficiency of the cleaning process was determined from the normalized water permeability (NWP) after hydraulic cleaning was carried out at the end of each experiment.

**Figure 9:**
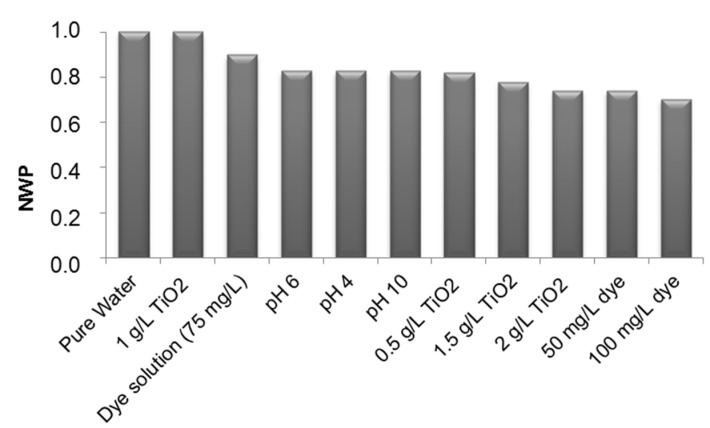
Evolution of normalized water permeability (NWP) for different experimental conditions.

According to results shown in Figure 9, it can be stated that after the treatment of the dye solution, the water permeability of the membrane decreased about 10%. The dye particles were adsorbed on the membrane surface and they only could be completely removed after a chemical washing.

Despite the increase of ∆P observed in the experiment carried out at pH 10, the water permeability remained constant at all of the studied pH. This phenomenon indicated that the fouling observed in the alkaline medium was only a physical process.

At the end of all experiments, the membrane water permeability was reduced about 30%.

## 4. Conclusions

For C.I. Disperse Red 73 dye, the photocatalytic treatment provided degradation in the range of 60% to 90% and COD removal from 70% to 98%.

The effect of pH in the photocatalytic degradation of DR73 was tested and shows that the highest dye degradation was achieved at pH 4 whereas the lowest was at pH 10, which is in accordance with photocatalyst charge. The experiments carried out at different photocatalyst loading indicated that dye degradation increased when the photocatalyst concentration increased. From 50 to 100 mg·L^−1^, the best results were obtained when the initial dye concentration was 50 mg·L^−1^. As expected, the photocatalytic dye degradation followed the Langmuir-Hinshelwood model.

Finally, the photocatalytic membrane treatment provided an uncolored permeate (dye concentration lower than 0.5 mg·L^−1^) with a low concentration of residual organic matter, which could be reused in new dyeing processes. This study thus demonstrates the feasibility of the coupling of photocatalysis and filtration for the treatment of azo dye wastewater.
